# A Six-Month Study of Pulmonary Cancer in Albanian Women

**DOI:** 10.5402/2013/824670

**Published:** 2013-03-04

**Authors:** Jolanda Nikolla, Milda Nanushi, Gentian Vyshka, Hasan Hafizi

**Affiliations:** ^1^Internal Diseases Department, Hygeia Hospital, Tirana 1005, Albania; ^2^University Hospital of Pulmonary Diseases, Tirana 1005, Albania; ^3^Biomedical and Experimental Department, Faculty of Medicine, University of Tirana, Tirana 1005, Albania

## Abstract

Lung cancer is a potentially lethal disease, whose prevalence in Albania is constantly increasing, especially in women. Early diagnosis is extremely important with regard to life expectancy and quality. The authors conducted a survey on the behaviour in a sample group of Albanian women diagnosed with primary and secondary lung cancers. A discussion upon diagnostic methods, smoking habits, histological type, Karnofsky performance status (KPS), and treatment modalities is made. The data collected by the authors suggest that nonsmokers formed the main group of lung cancer female patients. The most frequent histological type was adenocarcinoma. Mesothelioma was the most frequent of the secondary pulmonary lung cancers, followed from metastasizing breast cancer. Despite a generally good performance of the cases, the diagnosis of pulmonary cancer is delayed. The data collected could not find a convincing etiological role of tobacco smoking, but caution is needed, regarding the short time length of the study and the sustained number of participants.

## 1. Introduction

The incidence of lung cancer is constantly increasing, and this is a phenomenology that has raised concerns since decades, mostly important, because the disease has changed its manifesting modalities, and even the histological profile has undergone modifications [1–3]. On the other hand, if previously the age-specific parameter regarded mainly older patients, actually the trend of having more and more younger patients with lung cancer and with females suffering increasingly from the latter, is unrelenting [4]. Tobacco smoking habits have been accused to play a direct role in the fact that females are manifesting increasingly a higher incidence of lung cancer, although conclusions hardly converge [5–7]. 

Albeit that numerous professional settings have been related to higher incidence of lung cancer, tobacco smoking still remains the mostly discussed factor. It seems nevertheless that several years of exposure to active or passive smoking are necessary before modifications of the bronchial mucosa can be detected. Furthermore, correlating the manifestation of the adenocarcinoma of the lung with tobacco smoking has been continuously an issue of controversy, although recent and more large studies have still found a relation, but rather a smaller one, when compared with other histological types of lung cancer [8, 9].

In the present study, the authors have tried to describe the behaviour of a sample group of 54 Albanian females suffering from lung cancer and recruited for a diagnostic and therapeutic followup at the University Hospital of Pulmonary Diseases (UHPD) in Tirana, during the period from January to June 2011. Several factors are discussed, such as the age of the patients, smoking habits, Karnofsky score, histological type, and treatment modalities. Aiming to profile the actual trend of lung cancer in Albanian women, authors also evaluated the largely mentioned role of tobacco smoking in this sample. Other studies have been sketched on the same issue but focusing on other parameters, such as pain and other oncologic variables [10]. Also, models of studying smoking behaviour in this setting and group of patients have been proposed, mainly in regard to disease perception and behavioural changes related to the latter [11].

## 2. Materials and Methods

In a retrospective study, we have evaluated the behaviour of female patients diagnosed and treated for lung cancer. All patients were admitted in a university facility (UHPD) of Tirana, and cases were grossly divided in “primary” and “secondary” lung cancer. The female patients diagnosed with lung cancer during the period January–June 2011 and treated with the previously mentioned facility were all recruited consequentially to the present study, with a sample group having a total of 54 cases.

The majority of the cases were diagnosed through thoracic computerized tomography (CT), namely, 28 from the total of 54 females that formed the present sample of study. The rest of the cases were diagnosed through other methods, mainly bronchoscopy. Karnofsky Performance Status (KPS) was used as a standard score of measuring the ability of cancer patients to perform ordinary tasks of the everyday life activities. KPS is a scale ranging from 0 to 100, with a higher score meaning the patient to be more able to carry out everyday activities; such a scale has been proposed more than sixty years from now and yet has never lost its validity and utility [12]. The stadification of the lung cancer has been done according to the system TNM (tumor, node, and metastasis) of the year 2009, seventh update [13].

Self-reporting of the number cigarettes per day was used as a measure to consider the presence of smoking as a risk factor [14]. For the purpose of the study, female patients were divided in smokers and nonsmokers, with the latter group never smoking or smoking occasionally (less than two cigarettes per day in an irregular basis); strong smokers (consuming 1 pack per year, equivalent of smoking twenty cigarettes daily for at least one year) formed a minority of the sample (see results later).

## 3. Results

The sample group was formed from 54 females in total. 30 of them suffered from primary lung cancer (57%), and the rest of 24 female patients manifested a secondary lung cancer (43%).

The age profile of the patients is described in the Table 1. Although the majority of cases were above 51 years of age (28/54, i.e., more than half of the total), worth mentioning is the fact that we had 17 cancer patients (31%) aging from 31 to 40 years old. 

From the sample group we had 6 patients self-referring as smokers; “heavy smokers” that exceeded their smoke consumption by more than twenty cigarettes per day were a minority (two from six “smokers”). Surprisingly, 48 female patients diagnosed with lung cancer self-referred as being nonsmokers; thus we had a large majority (89%) of nonsmoking patients, forming part of a group diagnosed and treated for lung cancer.

Regarding the distribution of histological types of the primary lung cancer, we had a majority of adenocarcinomas (20 from a total of 30 cases with primary lung cancer); the overall histological data are included in the Table 2.

**Figure 1:**
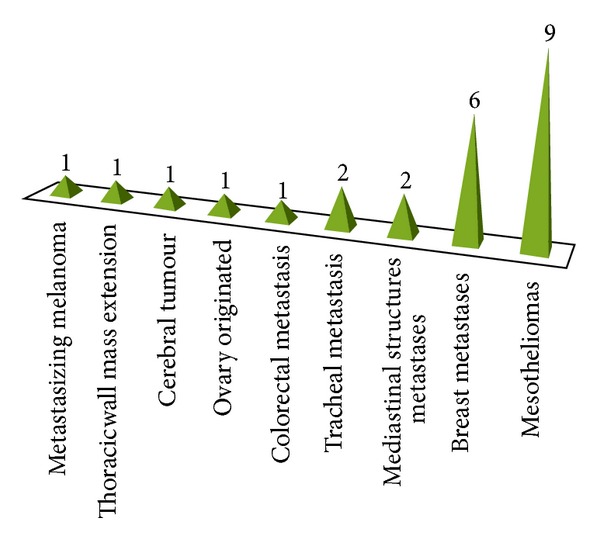
Graphic description of secondary lung cancer types, with their respective origins.

From the other subgroup with secondary lung cancer (24 cases in total) we had one case of metastasizing melanoma, one tumor of the thoracic wall extending per contiguity, one metastasizing cerebral tumour, one ovary-originated, and another one from colorectal origin; two cases had originated from trachea and another two from mediastinal structures. Breast metastasis and mesotheliomas formed the majority of the secondary lung cancer cases, respectively, with six and nine cases (see Figure 1 for the histological distribution of secondary lung cancer diagnosed in the sample group). 

In the following table (Table 3) we summarize the distribution of cases regarding the TNM staging, performance status (KPS), and the main modality of treatment the patients received.

From the data we gathered, we had the majority of cases presenting to a medical facility already in their fourth stage (TNM), although their performance status was generally good, which might comply with the surreptitious character of lung cancer, especially in nonsmoking women. 

The main modality of treatment offered was chemotherapy (45 out of a total of 54 patients); eight patients were operated and eventually half of them received thereafter chemotherapeutic treatment as well. We had no data regarding one case, which was treated in another facility, after the diagnosis was made.

## 4. Discussion

This is a descriptive and retrospective study, performed in a university facility, which is the only tertiary hospital dedicated to pulmonology in Albania. The data of the present study have a limited validity, since the number of patients (54 in total) and the time length of their recruitment (six months) are both parameters of a controversial significance, from the epidemiological point of view. 

In order to avoid confusions regarding the role of risk factors, we dichotomized the sample group merely in “smokers” and “nonsmokers”, without entering in details regarding the severity of tobacco smoking as a phenomenon. Such a severity must, for sure, play an important role in the genesis of the lung cancer; therefore it cannot be underemphasized. In fact, the definition of “nonsmokers”, “light smokers”, and “heavy smokers” are another subject of controversy; nonprofessional sources consider a light smoker as a person smoking less than five cigarettes per day, in confront to a “heavy” consumer, with more than twenty cigarettes (one pack) daily [15]. There are authors that have made much more meticulous separations of smokers in subgroups, such as “nonsmokers”, “heavy smokers”, “3-month quitters”, “6-month quitters”, “12-month quitters”, and “long-term quitters” [16]. A “heavy smoker index” has been proposed and applied in different studies [17]. Obviously, the scope of our study was more limited, and we merely divided patients into smokers and nonsmokers. 

From the data we gathered and the results presented herein, it is an impressive finding of a majority of “nonsmokers” suffering from lung cancer (48 out of 54 patients in the present sample, i.e., 89%). The question of secondary, second hand, or passive smoking will come out surely under these circumstances, and several sources have detailed the issue [18, 19]. In an obvious desperate attempt to control smoking in public environments, Albanian government passed an ad hoc bill, on November 2006, which forecasted severe fines applied to smokers; such fines in fact were never collected [20]. The law was stigmatized as the “forgotten law” from the media; but it is not excluded that other legislative or judicial acts might follow in the next future, since the era of litigation aiming to restrict tobacco exposure is surely arrived [21]. Hereby passive smokers and claims of liabilities related to health damages provoked to them are continuously upheld, especially in particular settings such as working places and so forth [22].

Another very important finding was the KPS, with Karnofsky scale scoring “high” in 59% of the cases. Such a good performance should have played some role in delaying the diagnosis, for we had on the other side, a very important number of cases staging at the fourth stage (TNM), with exactly 66% of the patients at the most advanced stage in the moment of diagnosis. Regarding the KPS we grouped the scores under the denominations of “low” when such a score was 10–40; “medium” when the score was 50–70 and “high” for the scores 80–100, such a grouping of scores has been applied from other sources as well [23, 24].

## 5. Conclusion 

The present study, while confirming the general medical concern regarding a possible increasing trend of lung cancer among Albanian women, raises two important issues. First, the fact that second-hand smoking role has to be scrutinized seriously and measured to be applied, when dealing with a majority of female lung cancer patients that self-referred as “nonsmokers”. Second, the fact that a large number of newly diagnosed lung cancer patients were already in advanced TNM stages albeit the performance status (KPS) was generally high requires a thorough revision of preventive medical policies and diagnostic procedures at the primary level of medical care that are obviously lacking the ability to early diagnose a potentially lethal disease. 

Since the initial clinical signs of this malignant disease are far from being specific or pathognomonic, guidelines for early referral and investigation are proposed and applied, with meaningful results [25]. This study tries to clarify some of the factors that have concerned Albanian clinicians recently regarding the trend of lung cancer among females. Albeit the sample is relatively small and the time length of the study is short, it is worth mentioning that parallel studies are difficult to find; paucity of sources has made comparative international conclusions, with Albania mentioned, rarely, if ever [26].

## Figures and Tables

**Table 1:** Age profile of the sample group.

20–30 years	1
31–40 years	17
41–50 years	8
51–60 years	14
>60 years of age	14

**Table 2:** Histological characteristics of the subgroup with primary lung cancer.

Histological type	Adenocarcinoma	Epidermoid lung cancer	Mixed type	Bronchioloalveolar carcinoma	Nonsmall cell lung carcinoma (NSCLC)	Total

Number of cases	20	3	3	2	2	30

**Table 3:** Tumour staging, performance status, and modalities of treatment.

	Number of cases	Percentages
Stage (TNM)		
I	5	9
II	4	8
IIIA	4	8
IIIB	5	9
IV	36	66

Total	54	100

Performance (KPS)		
Low	5	9
Medium	17	32
High	32	59

Total	54	100

Treatment (main modality)		
Operated	8	15
Chemotherapy	45	83
No data	1	2

Total	54	100

## References

[1] Gauderman WJ, Morrison JL (2000). Evidence for age-specific genetic relative risks in lung cancer. *American Journal of Epidemiology*.

[2] Tas F, Keskin S (2012). Age-specific incidence ratios of lung cancer (LC) in Turkey: LC in older people is increasing. *Archives of Gerontolology and Geriatrics*.

[3] Tanaka I, Matsubara O, Kasuga T, Takemura T, Inoue M (1988). Increasing incidence and changing histopathology of primary lung cancer in Japan: a review of 282 autopsied cases. *Cancer*.

[4] Beamis JF, Stein A, Andrews JL (1975). Changing epidemiology of lung cancer. Increasing incidence in women. *Medical Clinics of North America*.

[5] Ives A, Verne J (2010). What's happening to lung cancer in females?. *Thorax*.

[6] Boffetta P, Kreuzer M, Benhamou S (2001). Risk of lung cancer from tobacco smoking among young women from Europe. *International Journal of Cancer*.

[7] Lienert T, Serke M, Schönfeld N, Loddenkemper R (2000). Lung cancer in young females. *European Respiratory Journal*.

[8] Watson WL, Farpour A (1966). Terminal bronchiolar of “alveolar cell” cancer of the lung. Two hundred sixty-five cases. *Cancer*.

[9] Bracci PM, Sison J, Hansen H (2012). Cigarette smoking associated with lung adenocarcinoma in situ in a large case-control study (SFBALCS). *Journal of Thoracic Oncology*.

[10] Wilkie DJ, Keefe FJ, Dodd MJ, Copp LA (1992). Behavior of patients with lung cancer: description and associations with oncologic and pain variables. *Pain*.

[11] Browning KK, Wewers ME, Ferketich AK, Otterson GA, Reynolds NR (2009). The self-regulation model of illness applied to smoking behavior in lung cancer. *Cancer Nursing*.

[12] Karnofsky DA, Burchenal JH, McLeod CM (1949). The clinical evaluation of chemotherapeutic agents in cancer. *Evaluation of Chemotherapeutic Agents*.

[13] Rami-Porta R, Crowley JJ, Goldstraw P (2009). The revised TNM staging system for lung cancer. *Annals of Thoracic and Cardiovascular Surgery*.

[14] Perkins KA, Jao NC, Karelitz JL (2012). Consistency of daily cigarette smoking amount in dependent adults. *Psychology of Addictive Behaviors*.

[15] http://www.livestrong.com/article/228887-what-is-heavy-smoking/.

[16] Yongxin S, Wenjun D, Qiang W, Yunqing S, Liming Z, Chunsheng W (2013). Heavy smoking before coronary surgical procedures affects the native matrix metalloproteinase-2 and matrix metalloproteinase-9 gene expression in saphenous vein conduits. *The Annals of Thoracic Surgery*.

[17] Chabrol H, Niezborala M, Chastan E, de Leon J (2005). Comparison of the heavy smoking index and of the Fagerstrom test for nicotine dependence in a sample of 749 cigarette smokers. *Addictive Behaviors*.

[18] Adlkofer F (2001). Lung cancer due to passive smoking—a review. *International Archives of Occupational and Environmental Health*.

[19] Hackshaw AK (1998). Lung cancer and passive smoking. *Statistical Methods in Medical Research*.

[20] Law No.9636 http://www.qpz.gov.al/doc.jsp?doc=docs/Ligj%20Nr%209636%20Dat%C3%AB%2006-11-2006.htm.

[21] Miura M, Daynard RA, Samet JM (2006). The role of litigation in tobacco control. *Salud Pública de México*.

[22] Lesage FX, Deschamps F, Jurca D (2011). Illegal passive smoking at work. *Advances in Preventive Medicine*.

[23] http://www.hospicepatients.org/karnofsky.html.

[24] http://www.pennmedicine.org/homecare/hcp/elig_worksheets/Karnofsky-Performance-Status.pdf.

[25] Hippisley-Cox J, Coupland C (2011). Identifying patients with suspected lung cancer in primary care: derivation and validation of an algorithm. *British Journal of General Practice*.

[26] Voicu-Măceşeanu A, Nitu M, Olteanu M, Bică D (2007). Epidemiology of lung cancer. *Pneumologia*.

